# Multiple exposures to swine barn air induce lung inflammation and airway hyper-responsiveness

**DOI:** 10.1186/1465-9921-6-50

**Published:** 2005-06-02

**Authors:** Chandrashekhar Charavaryamath, Kyathanahalli S Janardhan, Hugh G Townsend, Philip Willson, Baljit Singh

**Affiliations:** 1 Immunology Research Group and Departments of Veterinary Biomedical Sciences, University of Saskatchewan, Saskatoon, S7N 5B4, Canada; 2 Large Animal Clinical Sciences, University of Saskatchewan, Saskatoon, S7N 5B4, Canada; 3 Vaccine and Infectious Disease Organization, University of Saskatchewan, Saskatoon, S7N 5B4, Canada

## Abstract

**Background:**

Swine farmers repeatedly exposed to the barn air suffer from respiratory diseases. However the mechanisms of lung dysfunction following repeated exposures to the barn air are still largely unknown. Therefore, we tested a hypothesis in a rat model that multiple interrupted exposures to the barn air will cause chronic lung inflammation and decline in lung function.

**Methods:**

Rats were exposed either to swine barn (8 hours/day for either one or five or 20 days) or ambient air. After the exposure periods, airway hyper-responsiveness (AHR) to methacholine (Mch) was measured and rats were euthanized to collect bronchoalveolar lavage fluid (BALF), blood and lung tissues. Barn air was sampled to determine endotoxin levels and microbial load.

**Results:**

The air in the barn used in this study had a very high concentration of endotoxin (15361.75 ± 7712.16 EU/m^3^). Rats exposed to barn air for one and five days showed increase in AHR compared to the 20-day exposed and controls. Lungs from the exposed groups were inflamed as indicated by recruitment of neutrophils in all three exposed groups and eosinophils and an increase in numbers of airway epithelial goblet cells in 5- and 20-day exposure groups. Rats exposed to the barn air for one day or 20 days had more total leukocytes in the BALF and 20-day exposed rats had more airway epithelial goblet cells compared to the controls and those subjected to 1 and 5 exposures (P < 0.05). Bronchus-associated lymphoid tissue (BALT) in the lungs of rats exposed for 20 days contained germinal centers and mitotic cells suggesting activation. There were no differences in the airway smooth muscle cell volume or septal macrophage recruitment among the groups.

**Conclusion:**

We conclude that multiple exposures to endotoxin-containing swine barn air induce AHR, increase in mucus-containing airway epithelial cells and lung inflammation. The data also show that prolonged multiple exposures may also induce adaptation in AHR response in the exposed subjects.

## Background

Respiratory diseases in agricultural workers are one of the earliest recognized occupational hazards [1]. Swine farmers work in confined buildings in close proximity to a large number of pigs and are exposed to toxic gasses such as ammonia and hydrogen sulfide, and to high levels of dust and endotoxins [2]. Exposure to such toxic bio aerosols including endotoxins in the barn air is a risk factor for the development of chronic respiratory symptoms and lung dysfunction [3-5]. Workers exposed to barn air report significantly higher frequencies of respiratory symptoms, cold, chest illness and pneumonia [2,3]. The severity of lung irritation and respiratory symptoms increases during winter and is also related to the number of working hours [6]. Single, 3–5 hour exposure of naïve, healthy, non-smoking subjects to swine barn air increases IL-6 in serum and IL-6 and IL-8 in nasal lavage and inflammatory cells in bronchoalveolar lavage fluid (BALF) [7,8]. Furthermore, pig barn dust stimulates IL-8 and IL-6 release from human bronchial epithelial cells *in vitro *[9]. Collectively, these data show that a single exposure to the barn air initiates acute lung inflammation.

Although swine barn workers are repeatedly exposed to barn air, majority of studies have focused on the acute pulmonary effects of single exposure [7,10]. Multiple exposures to barn air are linked to chronic lung inflammation including chronic bronchitis, decline in lung function and higher incidence of asthma [3,11,12]. Pig farmers with an average exposure history of 10.5 years and a daily exposure of 6.6 hours show significantly lower forced expiratory volume in one second (FEV_1_) and forced vital capacity (FVC) compared to unexposed control subjects [3]. Interestingly, acutely exposed naïve volunteers, show significantly more lung dysfunction, AHR, increase in cytokine levels and inflammatory cell numbers in blood and nasal lavage compared to the pig barn workers repeatedly exposed to the barn air [7,13,11]. These data suggest induction of an adaptive response in subjects repeatedly exposed to the barn air.

There is paucity of data on *in situ *cellular and molecular changes following multiple exposures to pig barn air. This is largely because of lack of an animal model to investigate the physiological impact of exposure to barn air. Therefore, we decided to undertake an *in vivo *single and multiple exposure study using rats to characterize cellular and molecular responses. We hypothesized that single and multiple exposures to swine barn air will induce lung inflammation and a decline in lung function. The data show that single and multiple exposures cause increase in AHR, inflammatory cells in BALF, mucus cells in the airways and lung inflammation.

## Methods

### Rats and treatment groups

The experimental protocols were approved by the University of Saskatchewan Campus Committee on Animal Care and experiments were conducted according to the Canadian Council on Animal Care Guidelines. Specific pathogen-free, six-week-old, male, Sprague-Dawley rats (Charles River Laboratories, Canada) were maintained in the animal care unit of Western College of Veterinary Medicine. Rats were randomly divided into four groups (n = 6 each). All personnel involved in collection and analyses of samples were blinded to the treatment groups.

### Exposure to swine barn air

We selected a regular commercial swine barn in the village of Aberdeen in Saskatchewan. The barn chosen for study had 60 dry sows and three boars. These pigs were fed with ground barley. Rat cages were hung from the barn ceiling at an approximate height of two meters above the floor. Groups of rats were exposed to barn air either for eight-hours for one-day, 5 days or for four cycles of 5 days (8 hours/day) each followed by 2 days in normal ambient air after every cycle. When rats were not exposed to the barn air, they were kept with the control animals in normal ambient air. Control rats were treated similarly except that they were not exposed to the barn air.

### Barn air sampling for endotoxin analysis

We sampled the barn air twice weekly to determine endotoxin levels as described previously [14]. Briefly, we collected airborne barn dust onto a pre-weighted, binder-free glass fibre inline filter (SKC Edmonton, Canada) hung at the level of rat cages. Barn air was drawn through the sampler (DuPont Air Sampler) for eight hours on each sampling day. The average flow-rate of the sampler was noted before and after each sampling period. Filters were desiccated before and after sampling. After weighing, the filters were placed in 50-mL polypropylene centrifuge tubes and were stored at 4°C until endotoxin analysis.

Endotoxin analysis was performed as described elsewhere [14]. Briefly, the filters with collected dust were washed individually in centrifuge tubes with 10 mL of sterile pyrogen-free water (DIN 00624721; Astra Pharma Inc; Mississauga, ON, Canada) followed by incubation for one-hour at room temperature in a sonicating water bath. Serial two-fold dilutions of the supernatant fluids were analyzed for Gram-negative bacterial endotoxin using an end-point assay kit as recommended by the manufacturer (model QCL-1000; Cambrex Bioscience Inc.; Walkersville, MD). The endotoxin standard (*Escherichia coli *O111:B4) was used in duplicate at four concentrations (0.1 to 1.0 endotoxin units (EU)/mL) in each assay to generate the standard curve. The lower detection limit was 0.1 EU/mL, which is equivalent to 1.0 EU per filter. The sampling time and flow rate were used to calculate the concentration of endotoxin in air (EU/m^3^).

### Viable microbial count

Viable microbial count was achieved using a six-stage viable cascade impactor (Graseby, Smyrna, GA). Air samples were collected from the vicinity of the rat cages hung from the ceiling of the barn by using a vacuum pump that was attached to the impactor capable of drawing air through the impactor at a rate of 1 ft^3^/ min (28.3 L/min). Six media plates of Tryptic Soy Agar with 5% sheep's blood were placed in the sampler and airborne microbes were directly collected onto 20 mL of media in 100 mm petri dishes. The air was drawn through the impactor for a duration of 15 seconds. The procedure was performed twice every week. The cascade impactor was cleaned thoroughly with 70% ethanol between each collection event. The plates were incubated at 37°C for 18–24 hours, and the colonies were counted using the positive-hole method correcting for microbial coincidence [15].

### Measurement of airway hyper-responsiveness

AHR was measured in awake control and exposed rats in response to increasing concentrations of methacholine (Mch) using head-out whole body plethysmography [16]. Air was supplied to the head and body compartments of the plethysmograph through a small animal ventilator (Kent Scientific, Litchfield, CT) and changes in respiratory airflow were monitored using a flow sensor (TRS3300; Kent Scientific, Litchfield, CT) linked via a preamplifier and A/D board (Kent Scientific) to a computer-driven real-time data acquisition/analysis system (DasyLab 5.5; DasyTec USA, Amherst, NH). The compartment of the plethysmograph, which accommodates the animal's head, was connected to an ultrasonic nebuliser (UltraNeb 99; Devilbiss Co., Somerset, PA) to expose the rats to Mch (Sigma Chemical Co. St. Louis, MO) [17,18]. Each rat was sequentially exposed to aerosols of saline alone (Mch 0 mg/ml) and then increasing doses of Mch diluted in saline (0.75, 1.5 and 3.0 mg/mL) and Flow@50%Tve1 (lung airflow at 50% of the expiratory tidal volume) was noted for saline and each of the Mch concentrations.

### Blood, bronchoalveolar lavage, tissue collection and processing

At the end of the exposure period, rats were euthanized (1 mg xylazine and 10 mg ketamine / 100 g) and blood, BALF and lung samples were collected. Blood was collected by cardiac puncture for differential and total leukocyte counts. BALF was collected by washing the whole lung with 3 ml of ice cold Hanks Balanced Salt Solution (Sigma Chemicals Co., St. Louis, MO). Three pieces from each lung lobe (left and right) were fixed in 4% paraformaldehyde for 16 hours and embedded in paraffin for light microscopy. Haematoxylin and eosin stained sections were used for histopathological evaluation of pulmonary inflammation.

### Quantification of mucus-producing cells

Mucus-producing goblet cells were quantified in lung sections stained with Periodic-acid Schiff (PAS) reagent [19]. Images were captured with the 20× objective lens of an Olympus microscope (Olympus BH2) connected to a digital camera (DVC Digital Camera, Diagnostic Video Camera Company, Austin, TX 78736-7735). The images were analysed using image analyses software (Northern Eclipse, version 6; Empix Imaging Inc., Mississauga, ON, Canada). Only those bronchi with a length to width ratio of less than 2.5 were selected for counting PAS-positive cells so as to minimize the error that might arise from tangential sectioning [20]. The PAS-positive goblet cells were counted manually and normalized to the length of the bronchial epithelial perimeter on the basal side, and expressed as the number of PAS-positive cells per mm of basement membrane.

### Immunohistochemistry

Lung sections were processed for immunohistochemistry as described previously [21]. Briefly, the sections were deparaffinized, hydrated and incubated with 5% hydrogen peroxide for 30 minutes to quench endogenous peroxidase, treated with pepsin (2 mg/ml in 0.01 N HCl) for 45 minutes to unmask the antigens and blocked with 1% bovine serum albumin for 30 minutes. Sections were incubated with primary antibodies against rat macrophage (1:400; ED-1, Serotec Inc. NC, USA) or monoclonal mouse anti-human smooth muscle actin (1:50; clone 1A4; DAKO A/S, Denmark), followed by appropriate biotinylated or horseradish peroxidase (HRP)-conjugated secondary antibodies (1:150; DAKO A/S, Denmark). Sections incubated with biotinylated antibodies were incubated with HRP conjugated streptavidin (1:300, DAKO A/S, Denmark) before color development. The reaction was visualized using a color development kit (VECTOR-VIP, Vector laboratories, USA). Controls consisted of staining without primary antibody or with isotype matched immunoglobulin instead of primary antibody.

### Quantification of macrophages and airway smooth muscle

ED-1 positive macrophages in the septa were counted in 20-high power fields (using 40× objective covering an area of 9.6 mm^2^). For smooth muscle quantification, a method described by Leigh *et al. *[22] was followed with a slight modification. A line was drawn along the outer border of the positively stained smooth muscle area and total stained area within that circle was measured using Northern Eclipse image analyses software. Next, a similar line was drawn along the inner border of the airway smooth muscle area to demarcate and measure the stained area. Stained area within the line drawn along smooth muscle inner border was deducted from the stained area within line drawn along smooth muscle outer border, to obtain the total stained area of airway smooth muscle. This total stained area of airway smooth muscle was normalized to the length of the outer perimeter of the airway smooth muscle, and results were expressed as, smooth muscle stained area in mm^2 ^per mm of airway smooth muscle perimeter.

### Statistical analyses

All data were expressed as mean ± SD. Group differences were examined for significance using one-way analysis of variance or two-way repeated measures analysis of variance with Fishers LSD as *post hoc *test (Sigma Stat Version 2.0, SPSS Inc., Chicago, IL 60611). Significance was established at P < 0.05.

## Results

### Barn air characterization

The mean endotoxin concentration in the swine barn air for the period of exposure was 15361.75 ± 7712.16 EU/m^3 ^of air. The amount of endotoxin in air samples from the room where control animals were kept (normal ambient air) was below the level of detection. The levels of endotoxin in the barn air in our study are much higher than those reported by other researchers [23,3]. The total viable aerobic bacterial counts in the barn air during the exposure period are shown in Table 1. Air samples collected from the room where control rats were kept did not yield any bacterial colonies.

**Table 1:** The total, respirable and non-respirable aerobic viable bacterial count (CFU/m^3 ^of air sampled) from the barn air

**Classification**	Viable aerobic bacterial count × 10^4 ^(CFU/m^3 ^of sampled air)*
Total	12.10 ± 8.47
Respirable	4.85 ± 4.97
Non-respirable	7.26 ± 7.50

* Viable bacterial counts are expressed as Mean ± SD.

### Airway hyper-responsiveness (AHR)

**Figure 1:**
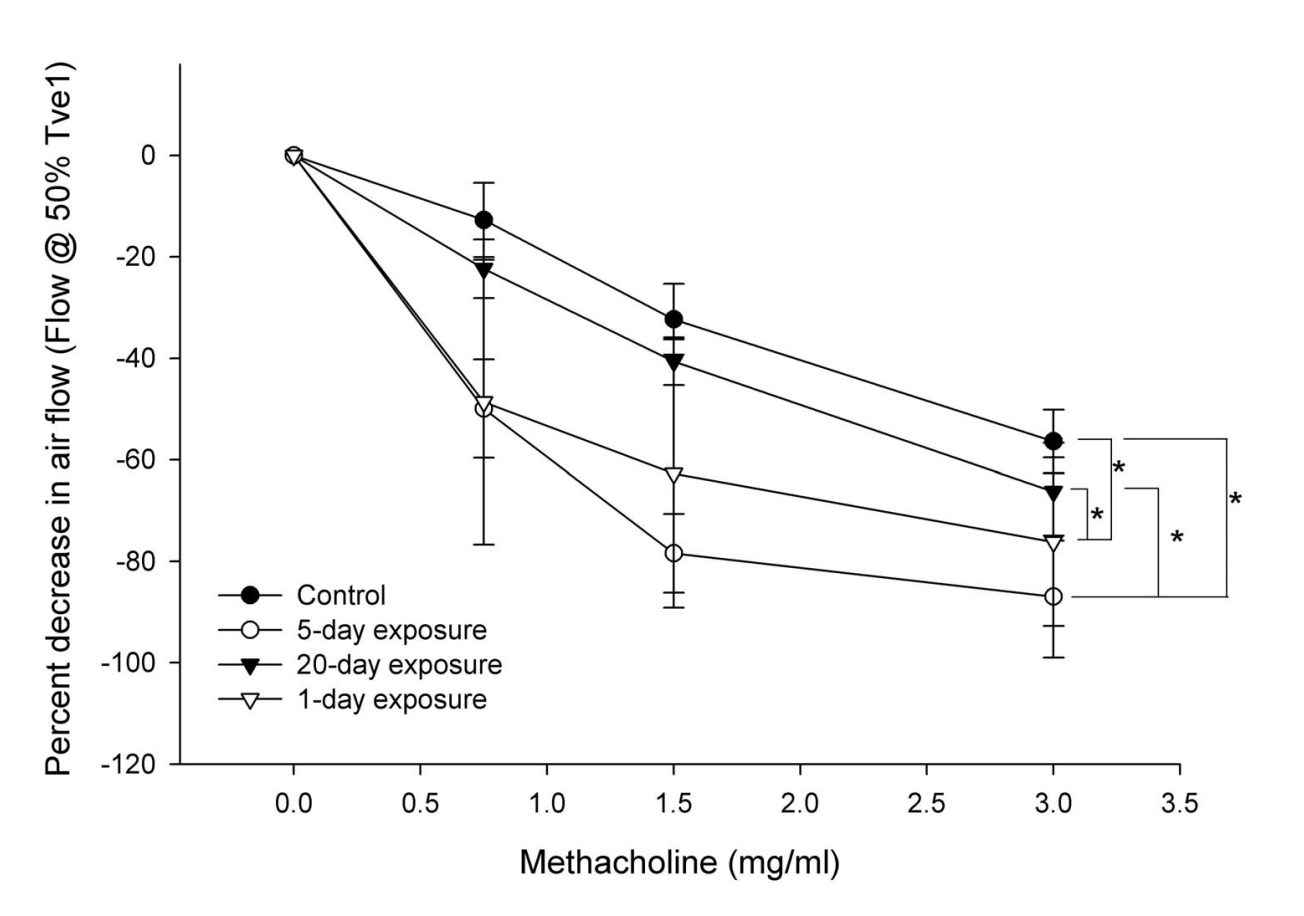
**Airway hyper-responsiveness. **Airway hyperresponsiveness to methacholine challenge in rats was measured using a whole-body head-out plathysmograph. Compared to controls, both 1-day and 5-day (P < 0.001) exposed rats showed increased airway hyperresponsiveness. Compared to 20-day exposed rats, 5-day (P = 0.001) and 1-day (P = 0.014) exposed rats showed increased airway hyper-responsiveness There was no difference between control and 20-day exposed (P = 0.207) and 1-day and 5-day exposed (P = 0.249) rats. *: Significantly different from other groups as indicated by line/s.

Inhalation of increasing concentrations of Mch caused decrease in airflow (Flow@50%Tve1) indicating airway reactivity and broncho-constriction. The data showed group differences in percent decrease in Flow@50%Tve1 (Figure 1; P < 0.001). Both 1- and 5-day exposed rats showed increased AHR compared to controls (P < 0.001) and 20-day exposed (P < 0.05). However, there were no differences in AHR between the control and 20-day exposed (P = 0.207) and 5-day and 1-day (P = 0.249) exposed rats.

### BALF cell counts

**Figure 2:**
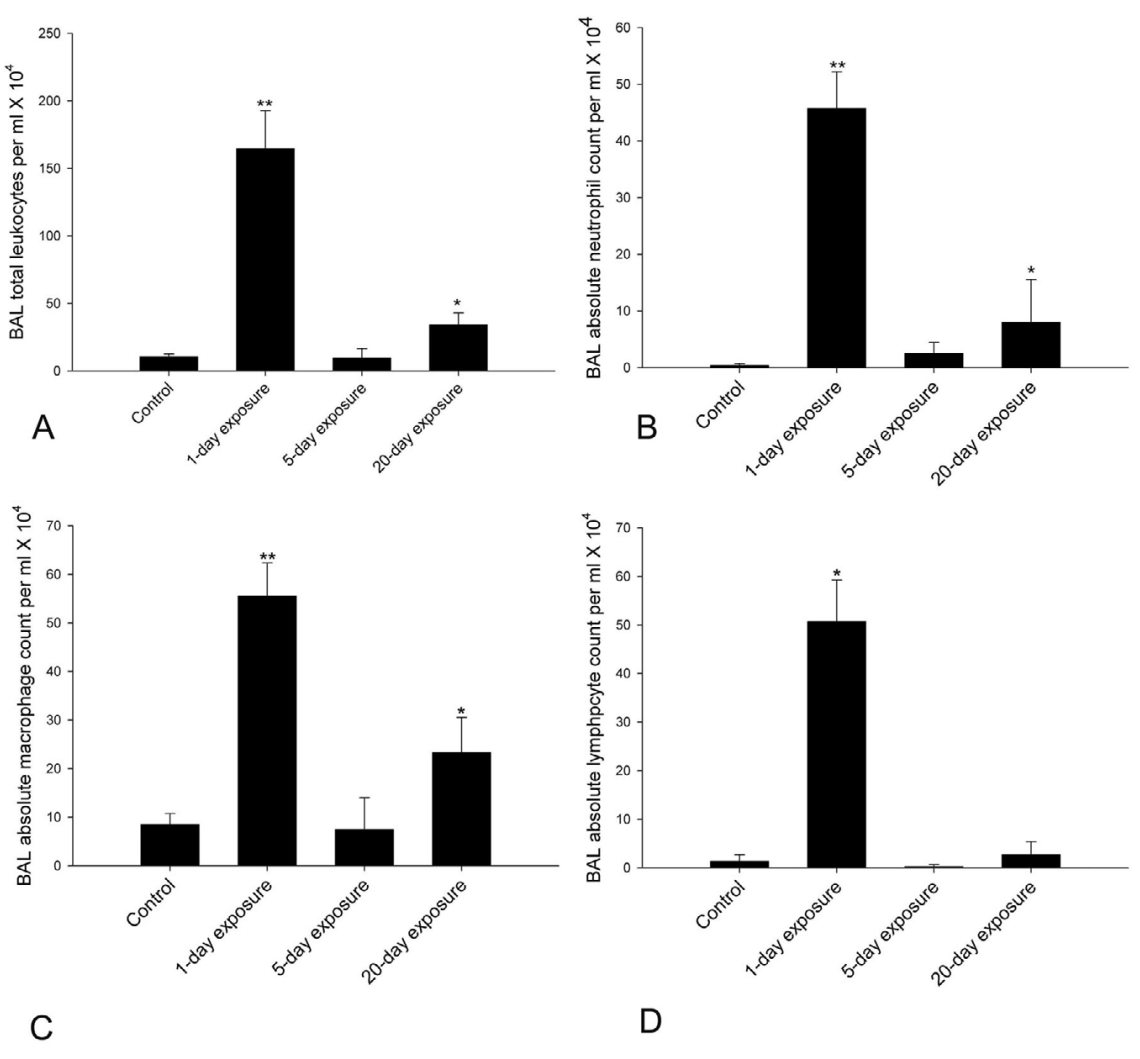
**Total and differential leukocytes in the bronchoalveolar lavage fluid. **Bronchoalveolar lavage was performed on the whole lung using 3 ml of cold HBSS. Cells were counted using a hemocytometer. Cytospins were prepared from BAL fluid and cells were differentiated with Wright's staining. 2A. BALF total leukocyte counts. BALF total leukocytes were different among the four groups (P < 0.001). Compared to controls, 5-day and 20-day exposed, 1-day exposed rats showed increased numbers of BALF total leukocytes (P < 0.001). Rats exposed for 20 days showed increased numbers of BALF total leukocytes when compared to controls (P = 0.01) and 5-day (P = 0.008) exposed rats. 5-day exposed rats did not differ from controls in their BALF total leukocyte numbers (P = 0.932). ** Significantly different from control, 5-day and 20-day exposed rats and * significantly different from control, 1-day and 5-day exposed rats. 2B. BALF absolute neutrophil counts. BALF absolute neutrophil counts were different among the groups (P < 0.001). 1-day exposed rats showed higher BALF absolute neutrophils when compared to control, 5-day and 20-day exposed rats (P < 0.001). 20-day exposed rats showed higher BALF absolute neutrophil count when compared to control rats (P = 0.022). There was no difference between control and 5-day exposed (P = 0.538) and 20-day and 5-day exposed (P = 0.119) rats. ** Significantly different from control, 5-day and 20-day exposed rats and * significantly different from control. 2C. BALF absolute macrophage counts. BALF absolute macrophage count was different among the four groups (P < 0.001). BALF absolute macrophage count was higher in 1-day exposed when compared to control, 5-day and 20-day exposed rats (P < 0.001). 20-day exposed rats showed higher BALF absolute macrophage count when compared to control and 5-day (P < 0.001) exposed rats. There was no difference between control and 5-day exposed rats (P = 0.789). ** Significantly different from control, 5-day and 20-day exposed rats and * indicates significantly different from control and 1-day and 5-day exposed rats. D. BALF absolute lymphocyte count (Figure 2D). BALF absolute lymphocyte count was different among the four groups (P < 0.001). BALF absolute lymphocyte count was higher in 1-day exposed when compared to control, 5-day and 20-day exposed rats (P < 0.001). * Significantly different from other three groups.

There were differences in total leukocyte counts in BALF among the four groups (Figure 2A; P < 0.001). The one day exposure group had higher BALF total leukocytes compared to the control, 5-day or 20-day exposed rats (P < 0.001). The 20-day exposed animals contained higher numbers of total leukocytes than control (P = 0.01) and those exposed for 5 days (P = 0.008). BALF total leukocytes were not different between control and 5-day exposed rats (P = 0.932).

The increased BALF total leukocytes in single exposure group, compared to control, 5- and 20-day exposed rats, were characterised by increased absolute neutrophil, macrophage and lymphocyte numbers (Figure 2B-D, P < 0.001). Increased BALF total leukocytes in 20-day exposed rats were characterized by increased absolute neutrophil (from controls, P = 0.022) and macrophage (control and 5-day exposed rats, P < 0.001) numbers. BALF absolute eosinophil numbers did not differ among the four groups (P = 0.178).

### Blood cell counts

**Figure 3:**
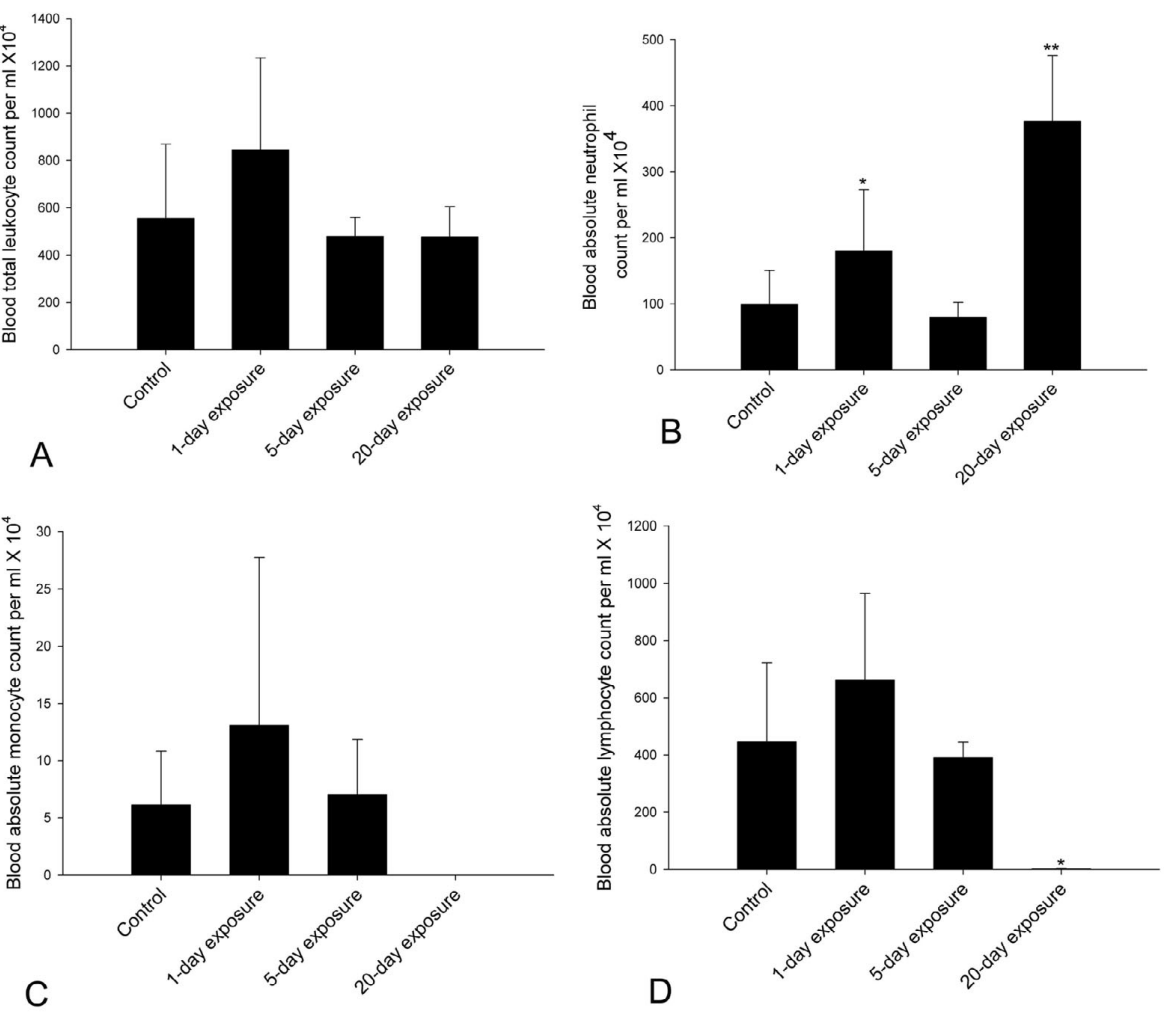
**Total and differential leukocyte count in blood. **Blood total leukocytes were counted using hemocytometer and smears were differentiated with Wright's stain. 3A. Blood total leukocyte count did not differ among the groups (Figure 3A; P = 0.090). 3B. Blood absolute neutrophils count was different among the four groups (Figure 3B; P < 0.001). 20-day exposed rats showed higher blood absolute neutrophils count when compared to control, 1-day and 5-day exposed rats (P < 0.001). 1-day exposed rats showed higher blood absolute neutrophil count when compared to 5-day exposed rats (P < 0.038). Both 1-day (P = 0.073) and 5-day exposed rats (P = 0.678) did not differ from controls. ** Indicate significantly different from control, 1-day and 5-day exposed rats and * indicate significantly different from 20-day and 5-day exposed rats. 3C. Blood absolute monocyte count did not differ among the four groups (Figure 3C; P = 0.122). 3D. Blood absolute lymphocyte count was different among the four groups (Figure 4D; P < 0.001). Compared to 20-day exposed, control (P = 0.003), 1-day (P < 0.001) and 5-day (P = 0.011) exposed rats showed increased numbers of blood absolute lymphocytes. * indicates significantly different from other three groups.

**Figure 4:**
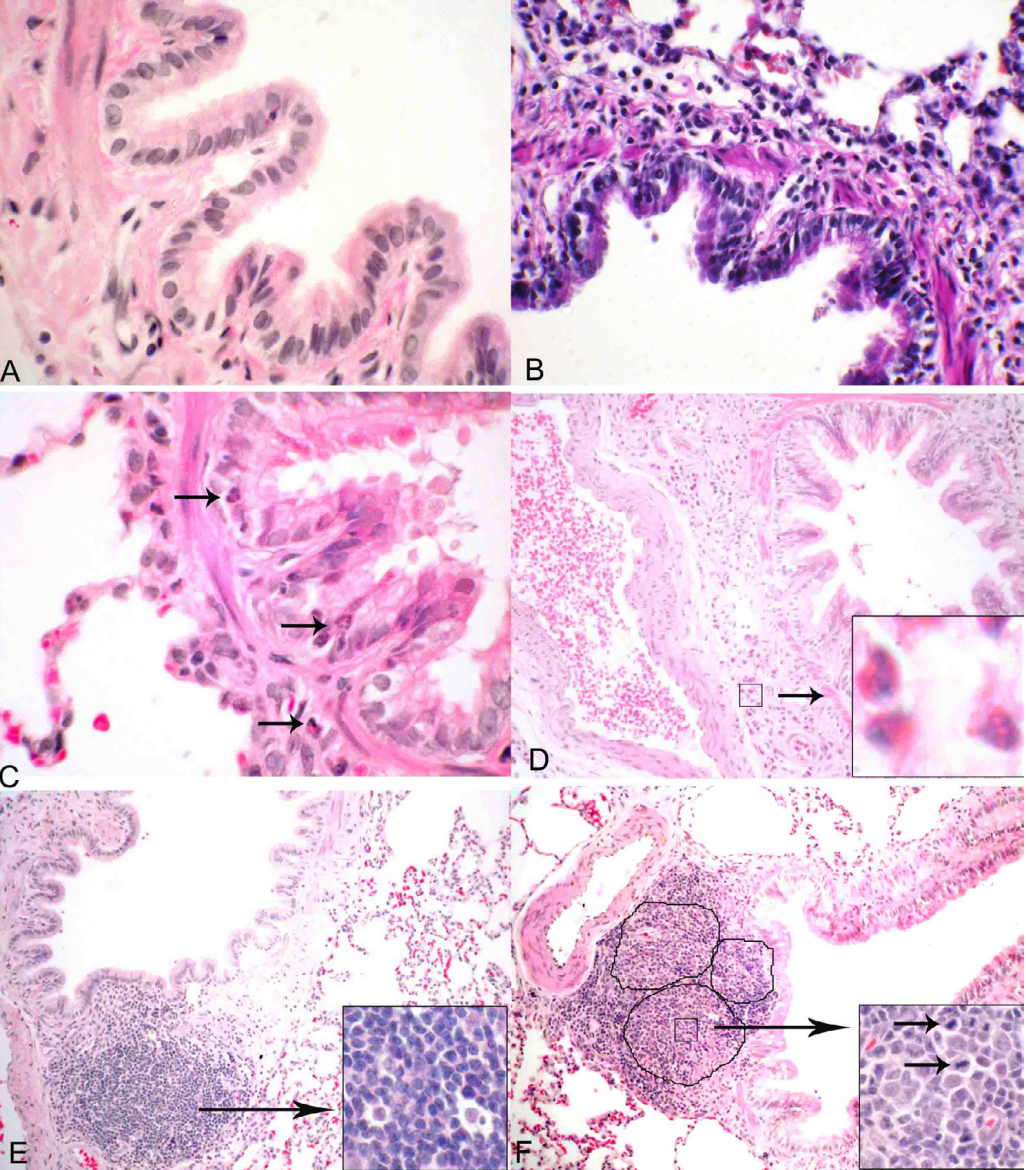
**Histopahtological evaluation of lung sections. **Histopathological changes in the lungs of swine barn air exposed and control rats were evaluated using hematoxylin and eosin stained sections. Control rat lungs (A) showed no inflammatory cell infiltration. Among the exposed groups, 1-day (B), 5-day (C) and 20-day exposed rats (not shown) showed peribronchiolar neutrophilic (C; arrows) and 5-day (D) and 20-day exposed (not shown) showed eosinophilic (D; arrows and inset) infiltration. Bronchus-associated lymphoid tissue (BALT) in control (E), 1-day and 5-day exposed (both not shown) appeared normal and had no germinal centers, whereas 20-day exposed rat lungs had activated BALT with germinal centers (F; outlined in black line) containing several mitotic cells (F; inset). *Original magnification *A-C: ×400; D-F: ×100; Insets: ×1000

There was no difference among the groups for total leukocyte counts (Figure 3A; P = 0.090). However, the absolute neutrophil numbers were different among the four groups (Figure 3B; P < 0.001). Rats exposed for 20 days showed higher absolute neutrophil numbers compared to the control and those exposed for 1 or 5 days (P < 0.001). Furthermore, rats exposed for 1 day showed higher blood absolute neutrophils when compared to 5-day exposed rats (P = 0.038). Blood absolute monocyte numbers did not differ among the four groups (Figure 3C; P = 0.122). Blood absolute lymphocyte numbers were different among the four groups (Figure 4D; P < 0.001). Compared to 20-day exposed, control (P = 0.003), 1-day (P < 0.001) and 5-day (P = 0.011) exposed rats showed increased numbers of blood absolute lymphocytes.

### Histopathology

Lung sections from control rats showed normal histology (Figure 4A) while those exposed for 1 day, 5 (Figure 4B-C) or 20 days (not shown) showed neutrophil infiltration into the lung tissue. Lung sections from 5-day (Figure 4D) and 20-day (not shown) exposed rats manifested perivascular and peribronchial eosinophil infiltration. Bronchus-associated lymphoid tissue (BALT) showed germinal centres and mitotic cells indicating BALT activation in rats exposed for 20 days (Figure 4F) compared to the controls (Figure 4E) or those subjected to 1 and 5 exposures (data not shown).

### Mucus cell quantification

**Figure 5:**
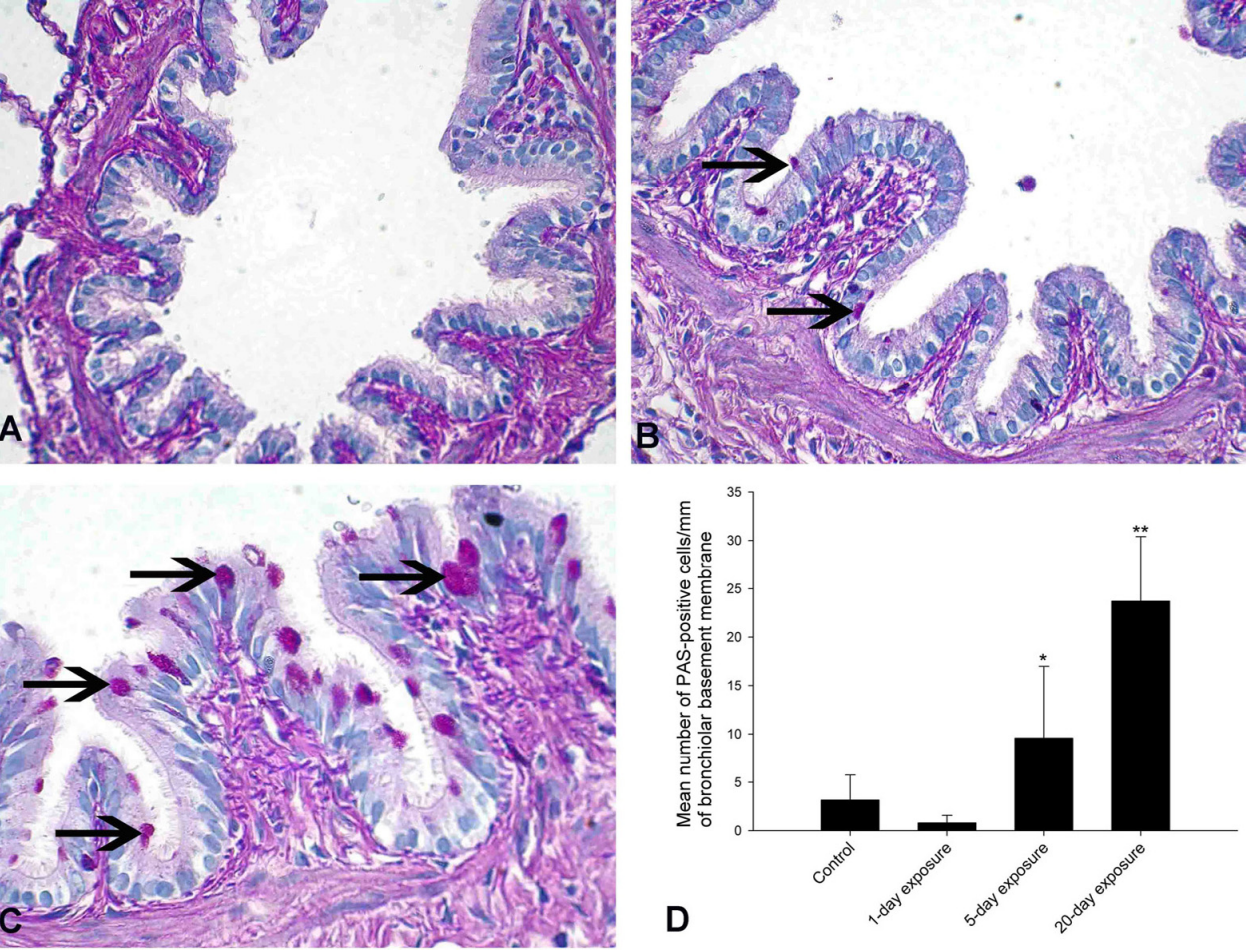
**Quantification of mucus producing cells in the airways. **Mucus producing goblet cells in the airways were quantified using PAS staining. Control rats showed no mucus producing cells in the bronchioles (A). 5-day exposed and 20-day exposed rats showed large number of mucus producing cells (B&C; arrows). Quantification of PAS-positive cells showed a significantly higher number of cells in 5-day and 20-day exposed rat lungs compared to the controls (5-day: P = 0.040; 20-day: P < 0.001) and one-day (5-day: P = 0.007; 20-day: P < 0.001) exposed rats (Figure D). Also, the increase in mucus producing cells was higher in 20-day exposed compared to 5-day exposed rat lungs (P < 0.001). Number of mucus producing cells did not differ between control and 1-day exposed rats (P = 0.435). *: Significantly different from control, 1-day and 20-day exposure. **: Significantly different from control, 1-day and 5-day exposure. The bars represent mean ± SD. *Original magnification *A-C; ×400

Because PAS method stains mucus as pink, it is commonly used as a method to identify mucus-containing cells (Figure 5). Morphometric data revealed more PAS-positive mucus-containing goblet cells in the airways of rats exposed for 5 or 20 days compared to the controls (5-day: P = 0.040; 20-day: P < 0.001) and 1-day (5-day: P = 0.007; 20-day: P < 0.001) exposed rats (Figure 5A-D). Furthermore, rats exposed 20 times contained more airway mucus cells compared to the 5-day exposure group (P < 0.001). There was no difference between control and 1-day exposed rats (P = 0.435).

### Quantification of ED-1 positive macrophages

**Figure 6:**
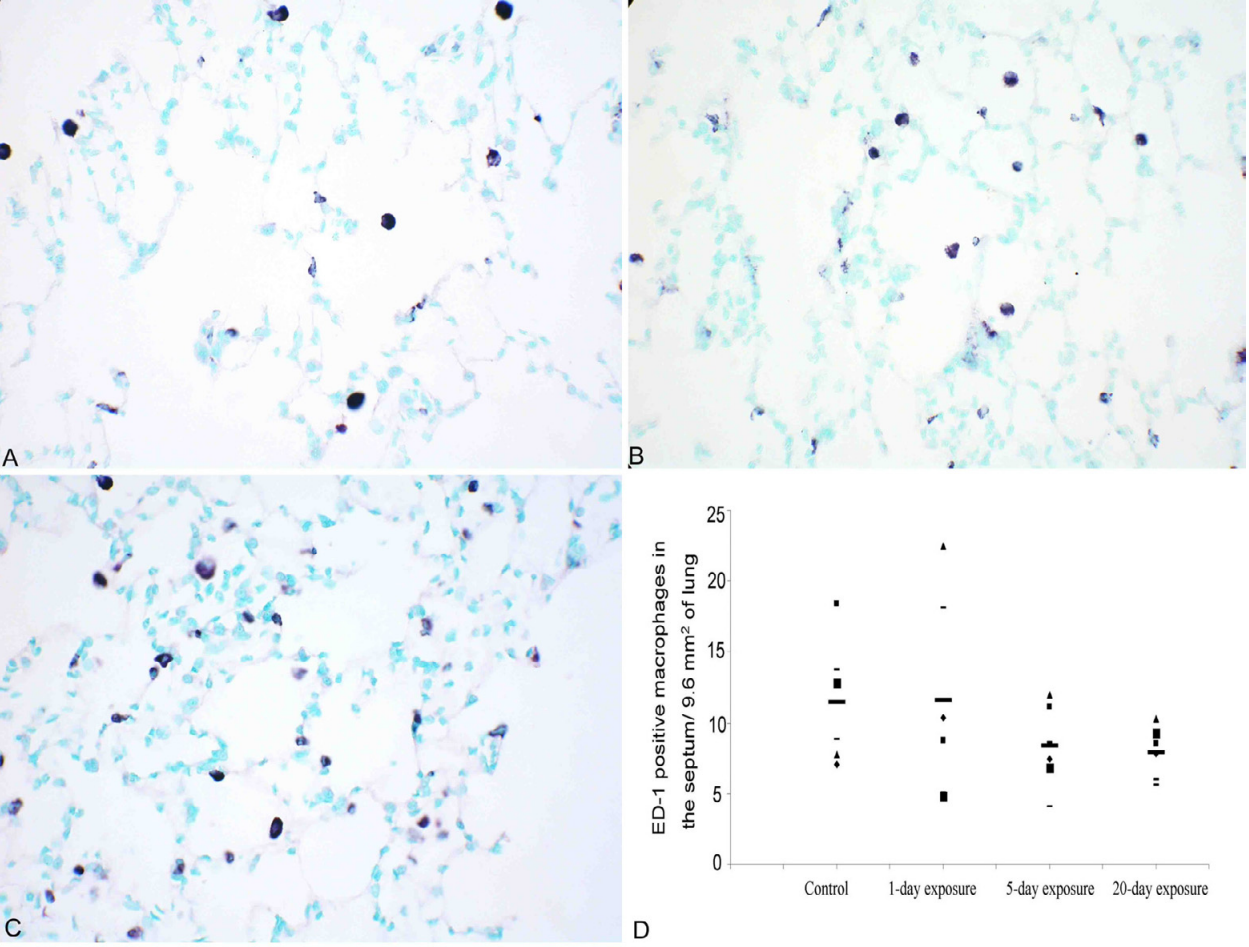
**Quantification of septal macrophages in the lung. **Macrophages were stained using ED-1 antibody. Lungs from control (A), 1-day (not shown in picture), 5-day exposed (B) and 20-day exposed (C) rats appeared to have similar numbers of septal macrophages. To confirm this we quantified ED-1 positive cells in the septum. D: Is a scatter plot showing number of ED-1 cells in the septum, in different groups. The horizontal bars in each group represent the mean for that particular group. There was no difference between the groups (P = 0.350). *Original magnification *A-C; ×400

The numbers of macrophages in the alveolar septa, stained with ED-1 antibody were not different among the four groups (Figure 6, P = 0.350).

### Immunohistochemical quantification for smooth muscle actin (SMA)

**Figure 7:**
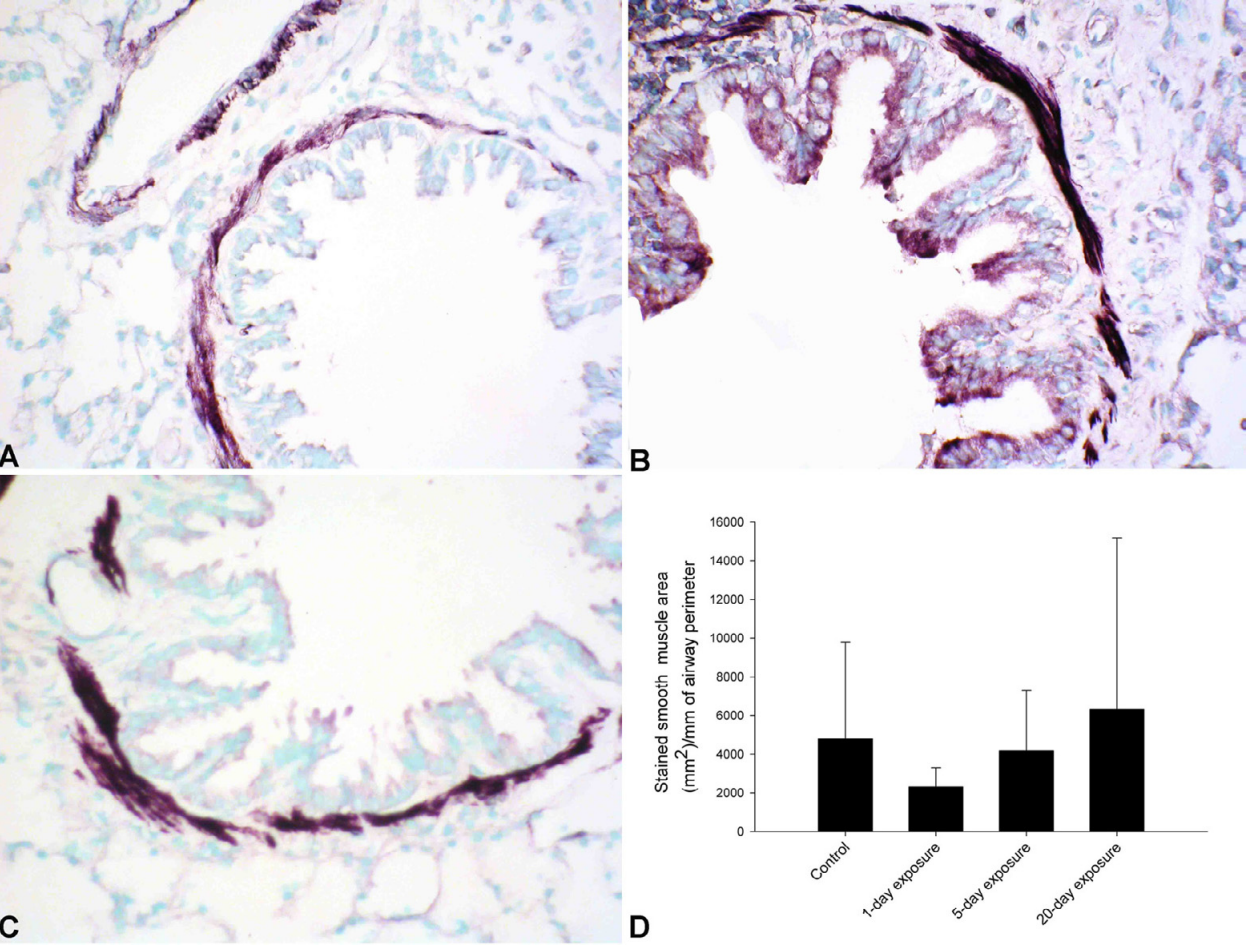
**Airway smooth muscle quantification. **The staining pattern for smooth muscle in controls (A), 1-day (not shown in picture), 5-day exposed (B) and 20-day exposed (C) rat lungs appeared similar. D. The stained area of smooth muscle around the bronchioles was measured using image analyses software. The area of smooth muscle was not significantly different between the groups (P = 0.681). *Original magnification *A-C; ×400

We used anti-human SMA antibody, which cross reacts with rat tissue to stain smooth muscles around the bronchi, bronchioles and blood vessels. Morphometric analyses showed no differences in smooth muscle area among the groups (Figure 7, P = 0.681).

## Discussion

We report *in vivo *and *in situ *data using an animal model on the effects of single and multiple exposures to the swine barn air. The data show that exposures to swine barn air induce an initial increase in AHR in one and five day exposed rats followed by an adaptive response in 20-day exposed rats; the 20-day group resembled the controls. Swine barn exposure induced lung inflammation in all the exposed groups characterized by infiltration of inflammatory cells, activation of BALT in 20-day exposed rats and an increase in mucus cells in the airway epithelium of 5- and 20-day exposed rats.

Our data show that one and five exposures to barn air induce significantly greater AHR in rats compared to 20 exposures and the unexposed. The AHR observed after 20 exposures was not different from controls. The precise mechanisms of increased AHR following one or five exposures to the barn air and an apparent adaptive response after 20 exposures remain incompletely understood. Previously, it was speculated that similar airway responses in the barn workers are initiated by the endotoxin present in the barn air [7,24]. It is likely that high levels of endotoxin in the barn air observed in our study are partially contributing to lung dysfunction induced in the exposed rats. Endotoxin in house dust has also been identified as a cause of lung dysfunction, which is characterized by increased AHR and inflammation [25]. Notwithstanding the cause of AHR following exposure to the highly complex barn air, there was amelioration of AHR in rats exposed for 20 days in conjunction with persistent inflammation. Previous data from a mouse model of allergic and IL-6 induced lung inflammation have shown dissociation between intensity of AHR and the lung inflammation [26,27]. Thus, our observations show that multiple exposures to barn air, which contains many toxic aerosols including endotoxins and ammonia, initially show an increase in AHR followed by an adaptive response. These data from exposed rats parallel the observations from barn workers who showed initial increase in AHR and decreased FEV1, FVC and mid-expiratory flow (FEV 25–75) followed by an adaptation indicated by less severe AHR [28,29]. Based on the similarity in lung responses following exposure to the barn air, the rat may be a good model to investigate *in vivo and in situ *cellular and molecular aspects of lung dysfunction in pig barn workers.

Rats, following single and 20 exposures, demonstrated more neutrophils and macrophages in their BALF. Rats exposed 20 times showed activation of BALT compared to the control and those exposed for 1 or 5 times indicating a progression towards chronic inflammation. BALT activation similar to that observed in our study has been reported in chronic bacterial infection [30,31], and following exposure to endotoxin and diesel exhaust [32,33]. Lung sections from all the exposed groups contained perivascular and peribronchial infiltration of inflammatory cells. It is well established that inflammatory cells are recruited in response to expression of adhesion molecules and chemoattractants on activated cells [34]. We believe that high levels of endotoxins measured in our study, in addition to other toxic aerosols such as ammonia, in the barn air may have activated expression of adhesion molecules and chemoattractants, such as IL-8, to promote recruitment of inflammatory cells [35-38].

Lung sections from rats exposed to the barn air for 20 days contained more mucus-containing goblet cells in the airway epithelium compared to the controls, 1 day and five day exposure group. Chronic LPS exposure [39] and many chronic respiratory diseases [40] present mucus hyper-secretion as a hallmark feature of airway inflammation. Such an increased mucus production in the airways is associated with reduced airway caliber, occlusion of small airways, reduced FEV1 [40], impaired gas exchange and compromised muco-ciliary clearance [41]. Our experiments do not identify the causative agent or the mechanisms of increase in mucus-containing goblet cells in the lungs of exposed rats. However, there are some possibilities. First, neutrophilic inflammation, such as one observed in the rats exposed to the barn air, has been shown to increase expression of epidermal growth factor and mucus synthesis [42]. Second, elastase released from activated neutrophils is known to stimulate degranulation of goblet cells and secretion of mucus [43]. Third, eosinophil recruitment, such as that observed in the lungs of 5- and 20-day exposed rats, is associated with goblet cell hyperplasia and increased mucus production in asthma and chronic obstructive pulmonary disease [44-46]. Lastly, chronic exposures to endotoxin, similar to those in our study, increase PAS-positive mucus cells [47,39]. These data show a causal relationship among exposures to the barn air, increased AHR, neutrophil and eosinophil recruitment, activation of BALT and goblet cell hyperplasia in the exposed rats.

Although we observed higher levels of endotoxins in the barn air, our study does not address precise mechanisms of BALT activation in the exposed rats. We believe that the inflammatory and increased AHR outcomes in our study are due to a combined effect of exposure to endotoxins and other toxicants such as ammonia [35,36,48] in the barn air. Although swine barn air contains both gram-positive and gram-negative bacteria [49,50], high levels of endotoxin in the study appear to be an indirect evidence for the presence of high-density of gram-negative bacteria in the barn air. We recorded higher levels of endotoxin in the barn air compared to those previously reported [3,23], which may be an outcome of husbandry practices as well as reduced ventilation in the winter season to conserve heat.

## Conclusion

Our data show that, single and multiple exposures to endotoxin rich-swine barn air induce lung inflammation characterized by infiltration of inflammatory cells, increased mucus positive-epithelial cells and activation of BALT in 20-day exposed rats. Furthermore, single and five exposures increased AHR. Because the barn air, in addition to endotoxins, contains dust, ammonia, microorganisms, aeroallergens [51], CO_2_, moulds [52], H_2_S [53], microorganisms and associated products such as bacterial cell wall, pig dander, fecal material and feed materials [54], more *in vivo *animal studies and detailed characterization of the barn air are needed to precisely identify the causative agents and their respective contributions to lung dysfunction and specific interactions of host genome and the environment.

## Competing interests

The author(s) declare that they have no competing interests.

## Authors' contributions

CC carried out the experiment, AHR, immunohistochemistry, image analyses, statistical analyses and drafted the manuscript. KSJ helped during the experiment, did histopathological evaluation, prepared figures and participated in manuscript preparation. HT participated in the statistical analyses. PW performed endotoxin analysis and helped to calculate total bacterial count in the barn air. BS conceived of the study, participated in its design and coordination as well as manuscript preparation. All authors have read and approved the final manuscript.

## References

[B1] Schenker MB, Christiani D, Cormier Y, Dimich-Ward H, Doekes G, Dosman JA, Schenker MB (1998). Respiratory health hazards in agriculture.

[B2] Asmar S, Pickrell JA, Oehme FW (2001). Pulmonary diseases caused by airborne contaminants in swine confinement buildings. Vet Hum Toxicol.

[B3] Zejda JE, Barber EM, Dosman JA, Olenchock SA, McDuffie HH, Rhodes CS, Hurst TS (1994). Respiratory health status in swine producers relates to endotoxin exposure in the presence of low dust levels. J Occup Med.

[B4] Zejda JE, Hurst TS, Rhodes CS, Barber EM, McDuffie HH, Dosman JA (1993). Respiratory health of swine producers: Focus on young workers. Chest.

[B5] Frevert CW, Warner AE (1999). Respiratory distress resulting from acute lung injury in the veterinary patient. J Vet Int Med.

[B6] Iversen M, Kirychuk SP, Drost H, Jacobson L (2000). Human health effects of dust exposure in animal confinement buildings. J Agric Saf Health.

[B7] Larsson K, Eklund AG, Hansson LO, Isaksson BM, Malmberg PO (1994). Swine dust causes intense airways inflammation in healthy subjects. Am J Respir Crit Care Med.

[B8] Dosman JA, Senthilselvan A, Kirychuk SP, Lemay S, Barber EM, Willson P, Cormier Y, Hurst TS (2000). Positive human health effects of wearing a respirator in a swine barn. Chest.

[B9] Romberger DJ, Bodlak V, von Essen SG, Mathisen T, Wyatt TA (2002). Hog barn dust extract stimulates IL-8 and IL-6 release in human bronchial epithelial cells via PKC activation. J Appl Physiol.

[B10] Palmberg L, Larsson BM, Sundblad BM, Larsson K (2004). Partial protection by respirators on airways responses following exposure in a swine house. Am J Ind Med.

[B11] Palmberg L, Larssson BM, Malmberg P, Larsson K (2002). Airway responses of healthy farmers and nonfarmers to exposure in a swine confinement building. Scand J Work Environ Health.

[B12] Pedersen B, Iversen M, Bundgaard LB, Dahl R (1996). Pig farmers have signs of bronchial inflammation and increased numbers of lymphocytes and neutrophils in BAL fluid. Eur Respir J.

[B13] Wang Z, Larsson K, Palmberg L, Malmberg P, Larsson P, Larsson L (1997). Inhalation of swine dust induces cytokine release in the upper and lower airways. Eur Respir J.

[B14] Kirychuk SP, Senthilselvan A, Dosman JA, Zhou C, Barber EM, Rhodes CS, Hurst TS (1998). Predictors of longitudinal changes in pulmonary function among swine confinement workers. Can Resp J.

[B15] ANDERSEN AA (1958). New sampler for the collection, sizing, and enumeration of viable airborne particles. J Bacteriol.

[B16] Neuhaus-Steinmetz U, Glaab T, Daser A, Braun A, Lommatzsch M, Herz U, Kips J, Alarie Y, Renz H (2000). Sequential development of airway hyperresponsiveness and acute airway obstruction in a mouse model of allergic inflammation. Int Arch Allergy Immunol.

[B17] Vijayaraghavan R, Schaper M, Thompson R, Stock MF, Alarie Y (1993). Characteristic modifications of the breathing pattern of mice to evaluate the effects of airborne chemicals on the respiratory tract. Arch Toxicol.

[B18] Vijayaraghavan R, Schaper M, Thompson R, Stock MF, Boylstein LA, Luo JE, Alarie Y (1994). Computer assisted recognition and quantitation of the effects of airborne chemicals acting at different areas of the respiratory tract in mice. Arch Toxicol.

[B19] Leigh R, Ellis R, Wattie JN, Hirota JA, Matthaei KI, Foster PS, O'Byrne PM, Inman MD (2004). Type 2 cytokines in the pathogenesis of sustained airway dysfunction and airway remodeling in mice. Am J Respir Crit Care Med.

[B20] Brass DM, Savov JD, Gavett SH, Haykal-Coates N, Schwartz DA (2003). Subchronic endotoxin inhalation causes persistent airway disease. Am J Physiol Lung Cell Mol Physiol.

[B21] Singh B, Rawlings N, Kaur A (2001). Expression of integrin avb3 in pig, dog and cattle. Histol Histopath.

[B22] Leigh R, Ellis R, Wattie J, Southam DS, De Hoogh M, Gauldie J, O'Byrne PM, Inman MD (2002). Dysfunction and remodeling of the mouse airway persist after resolution of acute allergen-induced airway inflammation. Am J Respir Cell Mol Biol.

[B23] Senthilselvan A, Zhang Y, Dosman JA, Barber EM, Holfeld LE, Kirychuk SP, Cormier Y, Hurst TS, Rhodes CS (1997). Positive human health effects of dust suppression with canola oil in swine barns. Am J Respir Crit Care Med.

[B24] Cormier Y, Duchaine C, Israel-Assayag E, Bedard G, Laviolette M, Dosman J (1997). Effects of repeated swine building exposures on normal naive subjects. Eur Respir J.

[B25] McKinley L, Kim J, Bolgos GL, Siddiqui J, Remick DG (2004). Reproducibility of a novel model of murine asthma-like pulmonary inflammation. Clin Exp Immunol.

[B26] DiCosmo BF, Geba GP, Picarella D, Elias JA, Rankin JA, Stripp BR, Whitsett JA, Flavell RA (1994). Airway epithelial cell expression of interleukin-6 in transgenic mice. Uncoupling of airway inflammation and bronchial hyperreactivity. J Clin Invest.

[B27] Kobayashi T, Iijima K, Kita H (2003). Marked Airway Eosinophilia Prevents Development of Airway Hyper-responsiveness During an Allergic Response in IL-5 Transgenic Mice. J Immunol.

[B28] Senthilselvan A, Dosman JA, Kirychuk SP, Barber EM, Rhodes CS, Zhang Y, Hurst TS (1997). Accelrated lung function decline in swine confinement workers. Chest.

[B29] Bessette L, Boulet LP, Tremblay G, Cormier Y (1993). Bronchial responsiveness to methacholine in swine confinement building workers. Arch Environ Health.

[B30] Rodriguez F, Ramirez GA, Sarradell J, Andrada M, Lorenzo H (2004). Immunohistochemical labelling of cytokines in lung lesions of pigs naturally infected with Mycoplasma hyopneumoniae. J Comp Pathol.

[B31] Iwata M, Sato A (1990). [A rat model of chronic bronchiolitis due to Pseudomonas aeruginosa--a histopathological study of bronchus-associated lymphoid tissue (BALT)]. Kansenshogaku Zasshi.

[B32] Ermert M, Ruppert C, Gunther A, Duncker HR, Seeger W, Ermert L (2002). Cell-specific nitric oxide synthase-isoenzyme expression and regulation in response to endotoxin in intact rat lungs. Lab Invest.

[B33] Ermert L, Ermert M, Merkle M, Goppelt-Struebe M, Duncker HR, Grimminger F, Seeger W (2000). Rat pulmonary cyclooxygenase-2 expression in response to endotoxin challenge: differential regulation in the various types of cells in the lung. Am J Pathol.

[B34] Lynch EL, Little FF, Wilson KC, Center DM, Cruikshank WW (2003). Immunomodulatory cytokines in asthmatic inflammation. Cytokine & Growth Factor Reviews.

[B35] Vogelzang PFJ, van der Gulden WJ, Folgering H, Heederk D, Tielen MJM, van Schayck CP (2000). Longitudinal changes in bronchial responsiveness associated with swine confinement dust exposure. Chest.

[B36] Donham KJ, Cumro D, Reynolds S (2002). Synergistic effects of dust and ammonia on the occupational health effects of poultry production workers. J Agromedicine.

[B37] Jagielo PJ, Thorne PS, Watt JL, Frees KL, Quinn TJ, Schwartz DA (1996). Grain dust and endotoxin inhalation challenges produce similar inflammatory responses in normal subjects. Chest.

[B38] Jagielo PJ, Thorne PS, Kern JA, Quinn TJ, Schwartz DA (1996). Role of endotoxin in grain dust-induced lung inflammation in mice. Am J Physiol.

[B39] Toward TJ, Broadley KJ (2002). Goblet cell hyperplasia, airway function and leukocyte infiltration after chronic lipopolysaccharide exposure in conscious guinea pigs: effect of rolipram and dexamethason. J Pharmacol Exp Ther.

[B40] Jackson AD (2001). Airway goblet-cell mucus secretion. Trends Pharmacol Sci.

[B41] Rogers DF (2001). Mucus hypersecretion in chronic obstructive pulmonary disease. Novartis Found Symp.

[B42] Kim JH, Lee SY, Bak SM, Suh IB, Lee SY, Shin C, Shim JJ, In KH, Kang KH, Yoo SH (2004). Effects of matrix metalloproteinase inhibitor on LPS-induced goblet cell metaplasia. Am J Physiol Lung Cell Mol Physiol.

[B43] Agusti C, Takeyama K, Cardell LO, Ueki I, Lausier J, Lou YP, Nadel JA (1998). Goblet cell degranulation after antigen challenge in sensitized guinea pigs. Role of neutrophils. Am J Respir Crit Care Med.

[B44] Williams TJ (2004). The eosinophil enigma. J Clin Invest.

[B45] Siergiejko Z (2003). [Bronchoalveolar lavage and induced sputum in asthmatic and COPD patient]. Pol Merkuriusz Lek.

[B46] Bocchino V, Bertorelli G, Bertrand CP, Ponath PD, Newman W, Franco C, Marruchella A, Merlini S, Del Donno M, Zhuo X, Olivieri D (2002). Eotaxin and CCR3 are up-regulated in exacerbations of chronic bronchitis. Allergy.

[B47] Vernooy JHJ, Dentener MA, van Suylen RJ, Buurman WA, Wouters EFM (2002). Long-term intratracheal lipopolysaccharide exposure in mice results in chronic lung inflammation and persistent pathology. Am J Respir Cell Mol Biol.

[B48] Sigurdarson ST, O'Shaughnessy PT, Watt JA, Kline JN (2004). Experimental human exposure to inhaled grain dust and ammonia: towards a model of concentrated animal feeding operations. Am J Ind Med.

[B49] Cormier Y, Tremblay GM, Mariaux A, Brochu G, Lavoie JP (1990). Airborne microbial contents in two types of swine confinement buildigns in Quebec. Am Ind Hyg Assoc J.

[B50] Clark S, Rylander R, Larsson L (1983). Airborne bacteria, endotoxin and fungi in dust in poultry and swine confinement buildings. Am Ind Hyg Assoc J.

[B51] Crook B, Robertson JF, Glass SA, Botheroyd EM, Lacey J, Topping MD (1991). Airborne dust, ammonia, microorganisms, and antigens in pig confinement houses and the respiratory health of exposed farm workers. Am Ind Hyg Assoc J.

[B52] Duchaine C, Grimard Y, Cormier Y (2000). Influence of building maintenance, environmental factors, and seasons on airborne contaminants of swine confinement buildings. AIHAJ.

[B53] Chenard L, Lemay SP, Lague C (2003). Hydrogen sulfide assessment in shallow-pit swine housing and outside manure storage. J Agric Saf Health.

[B54] Chang CW, Chung H, Huang CF, Su HJ (2001). Exposure of workers to airborne microorganisms in open-air swine houses. Appl Environ Microbiol.

